# Meditation as a Therapeutic Intervention for Adults at Risk for Alzheimer’s Disease – Potential Benefits and Underlying Mechanisms

**DOI:** 10.3389/fpsyt.2014.00040

**Published:** 2014-04-23

**Authors:** Kim E. Innes, Terry Kit Selfe

**Affiliations:** ^1^Department of Epidemiology, West Virginia University, Morgantown, WV, USA; ^2^Center for the Study of Complementary and Alternative Therapies, University of Virginia Health System, Charlottesville, VA, USA

**Keywords:** cognitive impairment, meditation, mind–body therapies, mood, sleep, stress, cellular aging, epigenetics

## Abstract

Alzheimer’s disease (AD) is a chronic, progressive, brain disorder that affects at least 5.3 million Americans at an estimated cost of $148 billion, figures that are expected to rise steeply in coming years. Despite decades of research, there is still no cure for AD, and effective therapies for preventing or slowing progression of cognitive decline in at-risk populations remain elusive. Although the etiology of AD remains uncertain, chronic stress, sleep deficits, and mood disturbance, conditions common in those with cognitive impairment, have been prospectively linked to the development and progression of both chronic illness and memory loss and are significant predictors of AD. Therapies such as meditation that specifically target these risk factors may thus hold promise for slowing and possibly preventing cognitive decline in those at risk. In this study, we briefly review the existing evidence regarding the potential utility of meditation as a therapeutic intervention for those with and at risk for AD, discuss possible mechanisms underlying the observed benefits of meditation, and outline directions for future research.

Alzheimer’s disease (AD), the most common form of dementia, is a chronic, progressive, brain disorder resulting in a loss of memory, reasoning, language skills, and the ability to care for one’s self (1). AD is the seventh leading cause of death in the US (2), affecting 5.2 million Americans at an estimated cost of $214 billion, figures that are expected to increase dramatically in the coming years (3, 4). AD affects quality of life for both the patient and the caregiver in profound ways. Many individuals with cognitive impairment become unable to engage in once loved activities that gave them a sense of purpose or pleasure (5). Behavioral and social skills may also deteriorate, which in turn, further increase risk for poor mental and physical health outcomes (6, 7). For example, neuropsychiatric symptoms are common in adults with AD (8–10), with up to 64% of AD patients experiencing disturbed sleep (11) and up to 87% suffering depressive symptoms (12, 13).

In most patients, AD develops slowly, often preceded years earlier by perceived and/or objective cognitive decline, offering a potential window for therapeutic intervention. First defined in the late 1990s and now widely recognized, mild cognitive impairment (MCI) is generally considered a transition phase between healthy cognitive aging and dementia (14, 15). Risk of progression to AD in those with MCI is very high, with an estimated 5–15% of those with MCI converting to AD each year (14–16), and up to 50% or more eventually progressing to AD (15). More recently, an even earlier harbinger of cognitive impairment, subjective cognitive impairment (SCI), has been associated with accelerated decline in cognitive function, an up to 4.5-fold increased risk for progression to MCI, and an approximately threefold elevated likelihood of developing AD (17–20). The annual conversion rate from SCI to MCI or dementia in otherwise healthy individuals has been estimated to be 7–10% (20, 21). While cognitive performance is only modestly reduced in those with MCI, and is in the normal range in those with SCI (22), recent research has shown cerebral spinal fluid markers of AD to be more common in adults with SCI and MCI, and to be associated with cognitive decline in those with MCI (23). In addition, prevalence of neuropsychiatric impairment is high in both these at-risk populations (8–10, 22), and, as discussed in more detail below, cognitive function appears strongly related, in a bidirectional manner, to anxiety, depression, and sleep disturbance (22, 24, 25). Up to 62% of MCI patients experience sleep disturbance (25, 26) and up to 83% suffer depressive symptoms (13), rates similar to those reported in AD (11, 12, 26). Similarly, adults with subjective memory complaints are significantly more likely to report symptoms of depression (19, 27) and anxiety (19). As detailed below, these mood and sleep disturbances lead, in turn, to accelerated cognitive decline and deterioration of both physical and mental health (12, 25, 28–31).

## Chronic Stress, Sleep Impairment, Mood Disturbance, and Cognitive Decline: A Vicious Cycle

Chronic stress can contribute to a vicious cycle that may not only have deleterious effects on health and cognitive function (32), but ultimately increase risk for institutionalization (29, 30). The chronic stress that often characterizes the lives of those with cognitive impairment, as well as their caregivers, has been linked to adverse changes in sleep (30), mood (33, 34), and immunological function (33, 35), and elevated risk for metabolic syndrome, cardiovascular disease (CVD), and mortality (36, 37). Chronic psychological stress can have profound effects on memory and behavior in persons both with and without cognitive impairment, and has been prospectively linked to increased risk for incident MCI and dementia in older adults, and to accelerated cognitive decline (38–40). Chronic stress leads to deleterious neuroendocrine and associated inflammatory changes, to suppression of IGF-1 and other neuroprotective factors, and to impaired synaptic plasticity, suppressed neurogenesis, reduced neuronal survival, and other adverse morphological and functional changes in the hippocampus, prefrontal cortex, and other brain structures; all these changes can profoundly affect mood, sleep, memory, and learning (41–45). A large body of experimental, clinical, and epidemiological research has also implicated chronic stress and associated sympathoadrenal activation in the etiology of hypertension, obesity, dyslipidemia, and other components of the metabolic syndrome, and in the development and progression of CVD, type 2 diabetes, depression, and related chronic disorders (41, 46). These disorders have, in turn, been shown to predict cognitive dysfunction, and to increase risk for the development and progression of AD (47–54). Autonomic and hypothalamic pituitary adrenal (HPA) axis dysfunction has also been linked directly to cognitive decline, and to adverse changes in brain structure and function. For example, HPA axis activation, manifested by elevated cortisol levels, has been associated with hippocampal volume loss and memory impairment in non-demented elders (41, 55).

As noted above, depression and other mood disorders are common in those with and at risk for AD, including adults with MCI and SCI (12, 13, 28, 56). Depressive symptoms and other distressful states have also been linked to significantly increased risk for diabetes, CVD, stroke, and the metabolic syndrome (57–59), and are a significant contributor to the profound reductions in quality of life reported by those with cognitive impairment (12, 31). Anxiety and depressive symptoms are also significant predictors of cognitive decline and incident cognitive impairment (60, 61). Moreover, in those with MCI, behavioral and psychological symptoms, including anxiety, depression, irritability, and apathy, are strong predictors of progression to AD (28, 62). In addition, mood disturbance can contribute not only to impairment of memory, but also to sleep disturbance, HPA axis dysregulation, and autonomic dysfunction and related pro-inflammatory changes, thus helping to promote a vicious cycle of adverse physiologic, neuroendocrine, and psychosocial changes that foster the development and progression of AD, CVD, and related chronic conditions (41, 63, 64).

Sleep disruption, also common in cognitively impaired adults (26), likewise has negative effects on health, functioning, and quality of life, and is a major reason for institutionalization (25, 29, 30, 65, 66). Sleep deficits are known to impair cognitive function in healthy populations (67, 68), to accelerate cognitive decline (29, 30), and to predict incident MCI and dementia (25). In addition, sleep disturbances have been strongly associated, in a bidirectional manner, with mood disorders (69) and autonomic dysfunction (70, 71), and can promote glucose intolerance, pro-inflammatory changes, dyslipidemia, obesity, and hypertension (70, 72, 73). Sleep impairment has likewise been linked to increased risk for incident type 2 diabetes and for CVD morbidity and mortality (41, 72–74), disorders that have, in turn, been significantly and prospectively linked to AD (75–78). The association of impaired sleep to chronic illness and related risk factors appears strongly reciprocal (72, 79).

## Alzheimer’s Disease and Cognitive Impairment: Need for New Prevention and Treatment Strategies

Despite decades of research, there is still no cure for AD. While a number of lifestyle factors have been linked to the subsequent development of this devastating disorder, effective therapies for preventing or slowing progression of AD in at-risk populations remain elusive (80), and there are no approved treatments for MCI or age-associated cognitive decline (22, 81). Given the high prevalence of chronic stress, sleep disturbance, and mood impairment in those with or at risk for cognitive impairment, the deleterious impact of these and related factors on health and cognitive function, interventions that specifically address these risk factors may hold promise not only for enhancing health and well-being, but also for slowing and possibly preventing cognitive decline in those at risk for AD. Of particular interest in this regard is meditation, an ancient mind–body practice that is gaining increasing favor throughout the western industrialized world as a means of reducing stress and improving both mental and physical well-being (82).

### Meditation: Health effects and therapeutic promise

Meditation has been broadly defined as “an intentional and self-regulated focusing of attention, whose purpose is to relax and calm the mind and body” (83). The studies included in this mini-review encompass a wide variety of meditative techniques, including mantra, mindfulness, and Kundalini meditation practices, among others. As indicated in recent systematic reviews by our group and other investigators, and by the growing body of original research on the health effects of meditation, there is mounting evidence that even brief meditative practices (5 days–8 weeks) may improve neuropsychological, metabolic, and clinical profiles in a range of populations (82, 84–87). For example, studies have shown meditation to reduce perceived stress (85, 88–90), anxiety (85, 88–91), and depressive symptoms (89–92), enhance quality of life (87, 92), decrease sleep disturbance (90, 93), improve several domains of cognition (90, 94, 95), reduce sympathetic activation and enhance cardiovagal tone (96–99), both acutely and long term in clinical as well as non-clinical populations (84, 100). A growing body of research also suggests that meditation promotes beneficial changes in CNS dopaminergic and other neurochemical systems (101, 102), and increases blood flow, oxygen delivery, and glucose utilization in specific regions of the brain associated with mood elevation, memory, and attentional processing, including the hippocampus, prefrontal cortex, and anterior cingulate gyrus (91, 95, 103–106). Long-term meditation practice has also been associated with cortical thickening and increased gray matter volume in brain regions involved in attentional performance, sensory processing, and interoception (103, 107, 108), apparently offsetting typical age-related cortical thinning and gray matter loss (108). In addition, recent research suggests that meditation programs can enhance immune response (109) and clinical outcomes (82, 85), and reduce blood pressure (85, 90, 100, 110), insulin resistance and glucose intolerance (97, 111), oxidative stress (84, 112), inflammation (93), and other related risk indices (84, 85). While research in cognitively impaired populations remains limited, findings from previous observational studies (113, 114) and two recent small clinical trials (90, 91, 95, 105) suggest that meditation practice may reduce stress, anxiety, depression, and blood pressure; improve cognition; promote beneficial changes in brain structure and function; and improve health outcomes in adults with memory disorders.

In addition to its many reported health benefits, meditation carries numerous practical advantages as a therapeutic intervention and health promotion method. It is a simple, economical, non-invasive therapy that is easy to learn and can be practiced even by very elderly, ill, or disabled individuals, including those suffering from cognitive impairment (82, 90, 95, 115). Requiring no special equipment and little in the way of professional personnel, meditation is a practice that is relatively easy to maintain at no cost, with several studies indicating excellent long-term adherence (84, 116, 117). Meditation practice typically brings immediate positive benefits, including feelings of relaxation and tranquility, and even short-term (5 day–6 week) meditation programs have been shown to result in significant improvement in mood, sleep, and other distressful symptoms (82, 84, 97, 110), helping to encourage continued practice. Several studies indicate that meditation programs may also reduce health care costs in both clinical and non-clinical populations (82, 118).

## Potential Underlying Mechanisms

**Figure 1:**
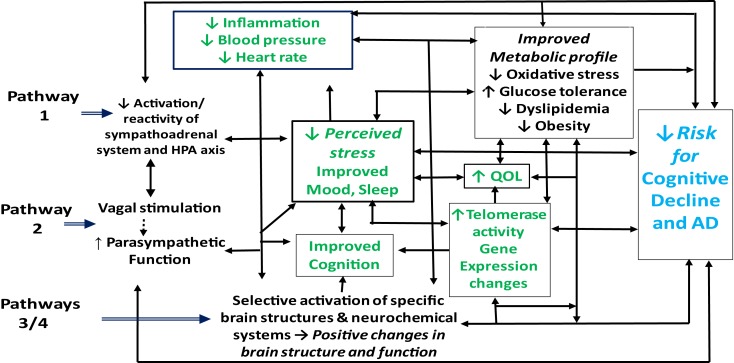
**Possible pathways by which meditation may improve health outcomes in adults with cognitive impairment**.

Although the mechanisms underlying the putative beneficial effects of meditation on cognitive, psychological, and physical health are not yet well understood, the observed changes likely occur through at least four pathways (41, 46, 84, 85, 101, 119). First, by reducing activation and reactivity of the sympathoadrenal system and the HPA axis and promoting feelings of well-being, meditation may alleviate the effects of stress, enhance sleep and mood, and foster multiple positive downstream effects on cognition, neuroendocrine status, neurological and metabolic function and related inflammatory responses (Figure 1, pathway 1). Second, meditation may enhance parasympathetic output, possibly via direct vagal stimulation, and thereby shift the autonomic nervous system balance from primarily sympathetic to parasympathetic, leading to positive changes in cardiovagal function, in mood, sleep, and energy state, and in related neuroendocrine, metabolic, and inflammatory responses, in turn, reducing risk for depression and cognitive decline (Figure 1, pathway 2). Third, findings of recent neuroimaging and neurophysiological studies (98, 106, 120) suggest that meditation, by selectively activating specific neurochemical systems and brain structures associated with positive mood, attention, and memory, may likewise promote beneficial changes in sympathetic/parasympathetic balance, in neurological structure and function, in affect and memory, and in related metabolic and inflammatory responses (Figure 1, pathway 3). Finally, findings of a recent study in dementia caregivers (121) suggest that meditation may also, by directly or indirectly stimulating increased telomerase activity, help promote telomere maintenance and buffer the effects of stress-induced cellular aging, thereby helping to preserve immune function and possibly reduce neuronal loss and other degenerative changes associated with aging and cognitive decline (122, 123). As discussed below, reductions in telomerase activity and telomere length have been linked to stress, depression, sleep loss, and cognitive impairment (99, 124–135) and shown to predict cognitive decline in both clinical and non-clinical populations (136, 137). Likewise, recent research in healthy adults (138–140), lonely older adults (141), and depressed dementia caregivers (142, 143) suggest that meditation may also buffer or reverse multiple stress-related changes in specific gene expression pathways implicated in the development and progression of AD, including those regulating oxidative stress, inflammation, cellular aging, and other factors contributing to impaired brain structure and function, and ultimately, to cognitive decline (144–150).

### Telomeres, inflammation, cognitive decline, and chronic stress: Possible benefits of meditation

An emerging body of literature suggests that both inflammation and telomere maintenance may be important factors in the pathogenesis of AD. Telomeres are DNA–protein complexes that protect the ends of chromosomes, and are essential for maintaining chromosomal integrity during replication (127, 151). The cellular enzyme telomerase acts to repair and replace the genetic telomeric material lost, helping to counteract the telomere shortening that occurs with age. There is growing evidence linking both shorter telomere length and lower telomerase activity with reduced survival and several age-related diseases (127, 151, 152), including AD (152–156) and suggesting that these telomeric alterations may mediate the degenerative changes associated with these conditions (131, 151, 157). Telomere shortening has been linked to cognitive impairment in several non-clinical populations (124–126) and shown in prospective studies to predict cognitive decline in post-stroke patients (136) and older community-dwelling women (137). Moreover, recent studies suggest that several lifestyle and environmental factors are important determinants of both telomere length and telomerase activity (127, 131, 152). Notably, these factors include chronic stress, depression, and impaired sleep, now emerging as powerful predictors of accelerated telomere shortening and reduced telomerase activity (99, 127–135). As indicated above, these deleterious changes may be buffered by meditative practices (99, 121, 158).

Telomere degradation may also be accelerated by chronic inflammation (156, 159, 160) which is, in turn, thought to be an important mechanism underlying cognitive decline and the pathogenesis of AD (54). Systemic inflammatory markers, including IL-6, TNF-alpha, and hsCRP, have been linked to loss of cerebral volume, and large population-based studies have consistently shown high blood levels of these inflammatory indices, to predict cognitive decline (54). Elevated inflammatory markers are also strongly associated, in a bidirectional manner, to chronic psychological stress, mood disturbance, sleep loss, and other distressful states (41, 72, 161–165). Emerging evidence suggests that meditative practices can not only reduce stress, improve sleep, and enhance mood, but may also decrease indices of systemic inflammation (121, 166, 167).

### Genomic changes in cognitive impairment, stress, and potential benefits of meditation

Genomic changes characterizing the inception and progression of AD is an active area of investigation (168–170). To date, over 180 genes distributed across the human genome have been directly or indirectly implicated in the pathogenesis of AD (168). Transcriptional profiling of blood mononuclear cells by microarray in those with AD have identified 19 upregulated and 136 downregulated genes (170, 171). While gene expression profiling in those with or at risk for AD is an emerging and still rapidly developing field of inquiry, recent studies suggest that multiple changes in gene expression may be important in AD progression (145, 147–150). These include alterations related to pro-inflammatory pathways (145), immune function (146), synaptic function (150), and regulation of apoptosis (146), oxidative stress (144) and other pathways (147, 148, 171); several of these changes have been linked to chronic psychological stress (139, 140, 144). Exploratory research in depressed dementia caregivers (142) and lonely older adults (141), as well as in healthy adults (138–140), suggests that the practice of meditation may lead to multiple beneficial changes in gene expression and may buffer or reverse adverse stress-related changes in transcriptional profiles. These include favorable alterations in several pathways linked to cognitive impairment, including those regulating cellular metabolism and aging, oxidative stress, immune function, inflammation, DNA repair, cell-cycle control, and apoptosis (138–143). However, while these preliminary findings suggest that meditative practices may help prevent, mitigate, or even reverse specific genomic changes implicated in cognitive decline, studies regarding the effects of meditation or other mind-body therapies on gene expression profiles in adults with memory loss are lacking.

### Summary

In brief, meditation may offer considerable promise as a safe and cost-effective intervention for reducing stress and for improving cognition, mood, sleep, and related outcomes in adults with or at risk for cognitive impairment. However, despite the apparent therapeutic potential of meditation for these populations, research remains sparse, and interpretation of existing studies is limited by small sample sizes, selection bias, and/or lack of appropriate control groups. Clearly, larger, rigorous randomized controlled trials are needed to establish the efficacy of meditation for improving cognitive function, stress, mood, sleep and related neuropsychosocial and physiological outcomes in adults with cognitive impairment, and to examine the long-term effects of meditation on cognitive decline and on the inception and progression of AD. Also needed are high-quality studies to assess the potential cost-effectiveness of meditation as a therapeutic intervention for those with or at risk for cognitive impairment, and to investigate potential underlying mechanisms, including changes in inflammatory markers, brain structure and function, cellular aging, and gene expression. If future studies show meditation to be effective in reducing stress and improving cognition and related outcomes in adults at risk for AD, it may offer a novel, safe, and low-cost approach to preventing or slowing cognitive decline in this population, and ultimately help reduce the significant health and economic burden associated with AD.

## Conflict of Interest Statement

The authors declare that the research was conducted in the absence of any commercial or financial relationships that could be construed as a potential conflict of interest.
